# Management of Complex Arrythmias: Optimal Timing of Catheter Ablation for Ventricular Tachycardia

**DOI:** 10.3390/jcm11175123

**Published:** 2022-08-31

**Authors:** Anurut Huntrakul, Jackson J. Liang

**Affiliations:** 1 Electrophysiology Section, Division of Cardiology, Cardiovascular Center, University of Michigan Medical Center, Ann Arbor, MI 48104, USA; 2 Division of Cardiovascular Medicine, Department of Medicine, Faculty of Medicine, Chulalongkorn University, King Chulalongkorn Memorial Hospital, Bangkok 10330, Thailand

The field of invasive cardiac electrophysiology has been rapidly advancing over the past several years. Novel tools, technologies, and numerous clinical trials have been actively developed to overcome the limitations of pre-existing treatment options. One increasingly utilized strategy for managing patients with ventricular tachycardia (VT) is radiofrequency catheter ablation, and the optimal timing for VT ablation remains unclear. 

Radiofrequency catheter ablation of VT is an effective treatment to reduce VT recurrence and implantable cardioverter–defibrillator (ICD) therapies. Despite this, patients with structural heart disease related VT are often referred for the procedure relatively late in their clinical course, especially patients who are managed by general cardiologists (non-electrophysiologists) in the community. Antiarrhythmic drugs (AADs) are often given as a first-line treatment in many patients, which may reduce VT recurrences and ICD therapies but can cause side effects and lead to long-term organ toxicities [1].

Previous observational studies demonstrated that early referral for VT ablation after the first episode of VT was associated with improved procedural success and higher VT-free survival [2,3]. The first large-scale randomized controlled trial (RCT) on early VT ablation was the Substrate Mapping and Ablation in Sinus Rhythm to Halt Ventricular Tachycardia (SMASH-VT) study, published in 2007 [4]. The authors reported a higher freedom from appropriate ICD therapy at 2-year follow-up with catheter ablation in patients with prior MI who had ICD therapy for single episode of VT or ventricular fibrillation (VF) compared with medical therapy. Later, The Ventricular Tachycardia Ablation in Coronary Heart Disease (VTACH) study, published in 2010, compared VT ablation plus ICD with ICD alone in patients with prior MI, reduced left ventricular ejection fraction (<50%), and had stable VT who qualified for secondary prevention ICD [5]. A longer time to VT recurrence and a higher freedom from recurrent VT/VF were found in ablation group. Then, the Ventricular Tachycardia Ablation versus Escalated Antiarrhythmic Drug Therapy in Ischemic Heart Disease (VANISH) trial, published in 2016, compared catheter ablation with escalation of AAD therapy (amiodarone or amiodarone plus mexiletine) in patients with ICM who had recurrent VT despite AAD therapy. A composite endpoint of death, VT storm, or appropriate ICD shock was lower in patients randomized to ablation, which was primarily driven by reduction in rates of appropriate ICD shocks and VT storm. All three previous studies did not demonstrate a mortality benefit of ablation. Importantly, there have been three recently published RCTs aiming to study the appropriateness and optimal timing of VT ablation, which are summarized below:

Does Timing of VT Ablation Affect Prognosis in Patients with an Implantable Cardioverter–Defibrillator? (PARTITA)

The PARTITA trial was a multicenter RCT assessing the effect of early VT ablation in patients with ischemic (ICM) or nonischemic cardiomyopathy (NICM) after their first ICD shock for VT [6]. A total of 47 patients were randomized 1:1 to immediate VT ablation (within 2 months from ICD shock) or continuation of standard therapy. The VT ablation strategy was to abolish all late potentials during sinus rhythm first and to perform activation mapping and ablation of induced VT if hemodynamically tolerated. Regarding AAD use, amiodarone was only allowed as a bridge to ablation after VT storm or for treatment of atrial arrhythmia. During a median follow-up of 2.4 years, the primary composite endpoint of all-cause mortality or heart failure hospitalization was significantly lower in ablation group (4% vs. 42%, HR 0.11, CI: 0.01–0.85, *p* = 0.034). Main secondary endpoints that occurred significantly less in the ablation group were all-cause mortality and recurrent ICD shocks for VT. The incidence of cardiac death and electrical storm, which are direct outcomes of a successful VT ablation were not different between two groups, possibly due to limited sample size.

Pan-Asia United States Prevention of Sudden Cardiac Death (PAUSE-SCD)

The PAUSE-SCD trial was a multicenter RCT which aimed to compare the efficacy of first-line VT ablation with conventional medical therapy at the time of ICD implantation in patients who had prior VT or inducible VT during electrophysiological study [7]. In this study, 133 patients with ICM, NICM, or arrhythmogenic cardiomyopathy (ARVC) were enrolled. After a median follow up duration of 31 months, the primary composite endpoint of VT recurrence (appropriate ICD shock or anti-tachycardia pacing), cardiovascular hospitalization, or all-cause death were less likely to occur in patients randomized to ablation versus medical therapy (45% vs. 59%, HR 0.58, CI: 0.35–0.96, *p* = 0.04). The difference was driven primarily by a reduction in rate of VT recurrence. In contrast to PARTITA, the mortality benefit was not demonstrated in this study. There were 5 (8.3%) procedural-related complications in the ablation group. One unique feature of PAUSE-SCD was that 30.6% of patients had NICM and 34.7% had ARVC while almost all other RCTs on VT ablation (other than PARTITA) were conducted in patients with ICM. As a result, 55% of patients in this study underwent epicardial approach ablation. The findings of PARTITA and PAUSE-SCD help to expand the evidence of early VT ablation in broader subgroups of patient with structural heart disease.

Substrate Ablation vs. Antiarrhythmic Drug Therapy for Symptomatic Ventricular Tachycardia (SURVIVE VT)

SURVIVE VT trial is a multicenter RCT which enrolled 145 patients with ICM and sustained VT who were randomized to a first-line VT ablation using endocardial substrate modification strategy versus AADs, which included amiodarone (86%) or sotalol (14%) [8]. The primary outcome was a composite of cardiovascular death, appropriate ICD shocks, heart failure hospitalization, or severe treatment-related complications. After 24-month follow up, the primary outcome occurred in 28.2% of patients in ablation group compared with 46.6% of patients in the AAD group (HR 0.52, CI: 0.30–0.90, *p* = 0.021). The difference of primary outcome was mainly driven by a reduction in severe treatment-related adverse events, with slow or incessant VT being the most frequent adverse events in AAD group. The rates of hospitalization for ventricular arrhythmia and VT storm were significantly lower in the ablation group, while the incidence of cardiovascular death and appropriate ICD shocks were similar between both groups. Regarding safety, seven patients (9.8%) in the ablation group experienced procedural complications. The key message of this trial is that endocardial substrate modification strategy for VT ablation is a safe and effective first-line treatment option in patients with ischemic VT. 

More recently, Ravi, et al. summarized in a meta-analysis the results from 11 studies (9 RCTs and 2 observational) comparing VT ablation with medical therapy [9]. Of 2126 patients (711 in the ablation arm, 1415 in the medical therapy arm), ablation was associated with lower risk of recurrent VT (RR 0.79, CI 0.67–0.93, *p* = 0.005), ICD shocks (RR 0.64, CI 0.45–0.89, *p* = 0.008), and cardiac hospitalizations (RR 0.76, CI 0.63–0.92, *p* = 0.005) but with no difference in mortality (*p* = 0.71).

In summary, based on the results of the aforementioned studies, supportive evidence for VT ablation as an early (even first line) therapy in patients with structural heart disease with sustained VT episodes is strengthened. VT ablation is a safe and effective treatment option when performed by experienced operators at high volume centers, and should be considered early in the clinical course in patients with VT.
